# Transcriptomic responses to 2,3,7,8-tetrachlorodibenzo-*p*-dioxin (TCDD) in liver: Comparison of rat and mouse

**DOI:** 10.1186/1471-2164-9-419

**Published:** 2008-09-16

**Authors:** Paul C Boutros, Rui Yan, Ivy D Moffat, Raimo Pohjanvirta, Allan B Okey

**Affiliations:** 1 Department of Pharmacology and Toxicology, University of Toronto, Toronto, Canada; 2 Department of Computer Science, University of Toronto, Toronto, Canada; 3 Department of Food and Environmental Hygiene, University of Helsinki, Helsinki, Finland

## Abstract

**Background:**

Mouse and rat models are mainstays in pharmacology, toxicology and drug development – but differences between strains and between species complicate data interpretation and application to human health. Dioxin-like polyhalogenated aromatic hydrocarbons represent a major class of environmentally and economically relevant toxicants. In mammals dioxin exposure leads to a broad spectrum of adverse affects, including hepatotoxicity of varying severity. Several studies have shown that dioxins extensively alter hepatic mRNA levels. Surprisingly, though, analysis of a limited portion of the transcriptome revealed that rat and mouse responses diverge greatly (Boverhof et al. Toxicol Sci 94:398–416, 2006).

**Results:**

We employed oligonucleotide arrays to compare the response of 8,125 rat and mouse orthologs. We confirmed that there is limited inter-species overlap in dioxin-responsive genes. Rat-specific and mouse-specific genes are enriched for specific functional groups which differ between species, conceivably accounting for species-specificities in liver histopathology. While no evidence for the involvement of copy-number variation was found, extensive inter-species variation in the transcriptional-regulatory network was identified; Nr2f1 and Fos emerged as candidates to explain species-specific and species-independent responses, respectively.

**Conclusion:**

Our results suggest that a small core of genes is responsible for mediating the similar features of dioxin hepatotoxicity in rats and mice but non-overlapping pathways are simultaneously at play to result in distinctive histopathological outcomes. The extreme divergence between mouse and rat transcriptomic responses appears to reflect divergent transcriptional-regulatory networks. Taken together, these data suggest that both rat and mouse models should be used to screen the acute hepatotoxic effects of drugs and toxic compounds.

## Background

In laboratory animals the environmental contaminant, 2,3,7,8-tetrachlorodibenzo-*p*-dioxin (TCDD, "dioxin") causes a wide variety of toxic effects, even at extraordinarily low levels of exposure (reviewed in: [1]), leading to concern about potential harm to health of humans exposed to these agents [2].

All major toxic effects of TCDD and related halogenated aromatic hydrocarbons appear to be mediated by a soluble protein, the aryl hydrocarbon receptor (AHR) [3,4]. The AHR resides quiescent in the cytoplasm in a multi-component complex until ligand-binding [5], which triggers a conformational change that leads to nuclear translocation. Once in the nucleus, the AHR and its heterodimerization partner, ARNT, can function either as a transcription-factor [6], a coactivator [7,8], or potentially as an E3 ubiquitin ligase [9]. As a transcription-factor, the AHR binds to a cognate response element termed the AHRE-I (Aryl Hydrocarbon Response Element I) [10]. As a coactivator, the AHR interacts with other DNA binding proteins including the estrogen receptor [8,11], Sp1 [12], and other uncharacterized transcription-factors [7].

Despite a fairly detailed molecular understanding of the manner in which the AHR regulates expression of genes, such as those encoding CYP1A enzymes [13], the linkage between the AHR molecular mechanism and biological manifestations of TCDD-induced toxicity remain, with few exceptions [14-16], elusive. The primary difficulty is that the AHR regulates – either directly or indirectly – hundreds of genes, often in tissue-specific patterns [17-24]. Many groups, including our own, have sought to associate specific genes with specific toxicologic outcomes by using intra-species models, where different strains vary in their sensitivity to TCDD (reviewed in [2] and [25]). However, these intra-species models are confounded by the extensive strain-to-strain variability observed in both mice [26] and rats (Boutros et al. submitted). Further, generating model systems where animals vary in sensitivity is a difficult problem lacking a general solution.

These challenges of intra-species models are not exclusive to TCDD but are common to the study of all drugs and toxicants in model organisms. One alternative approach is to compare the response to a drug or toxicant between closely-related species. For example, because mice and rats show largely similar phenotypic responses to TCDD one might hypothesize that transcriptional responses will be conserved. To test this hypothesis, Boverhof and coworkers compared the changes in mRNA abundance induced by TCDD in C57BL/6 mice and Sprague-Dawley rats using custom cDNA arrays [27]. They tested 3,087 orthologous genes and found only 33 that responded to TCDD in both species. This set of conserved, species-independent responses represents only 15.1% of rat genes and 12.8% of mouse genes that respond to TCDD. Thus approximately 85% of responses to TCDD are species-specific.

This has major implications for the use of mouse and rat as model organisms to study toxic responses, making it important to evaluate and extend this finding. We analyzed the transcriptional response to TCDD in TCDD-sensitive strains of mouse (C57BL/6) and rat (Long-Evans [*Turku/AB*]; L-E) using commercial oligonucleotide arrays. Our arrays contained 8,125 orthologous genes, allowing us to analyze a much larger portion of the transcriptome than the previous study. We confirmed that a very small fraction of TCDD-responsive genes show conserved patterns in mouse and rat liver, and that these represent specific GO categories. Further, we performed library-based and *de novo *transcription-factor binding-site analyses to rationalize the patterns of expression. Finally we validated our results with a gene-by-gene comparison to a public dataset.

## Results

We used mRNA expression microarray analysis to study the transcriptional response to TCDD in young adult male C57BL/6 mice and L-E rats. Eleven mice were used, five treated with corn-oil vehicle and six treated with 1000 μg/kg TCDD for 19 hours [28]. Eight rats were used, four treated with corn-oil vehicle and four treated with 100 μg/kg of TCDD for 19 hours. For both C57BL/6 mice [29,30] and L-E rats [31] the respective TCDD doses given in this study are about 5-fold higher than the single-dose LD_50 _values for male animals (Pohjanvirta et al. in preparation). At the 19-hour exposure time, we expect that most responses are likely to represent primary events although some early morphological manifestations of hepatic toxicity emerge within one day, at least in mice [27]. RNA extracted from each animal was hybridized to an oligonucleotide array. All microarray data have been deposited in the GEO repository (accessions GSE10769 and GSE10770). We carefully validated array quality; none were excluded (Additional file 1).

### Overall Transcriptional Response to TCDD

**Figure 1:**
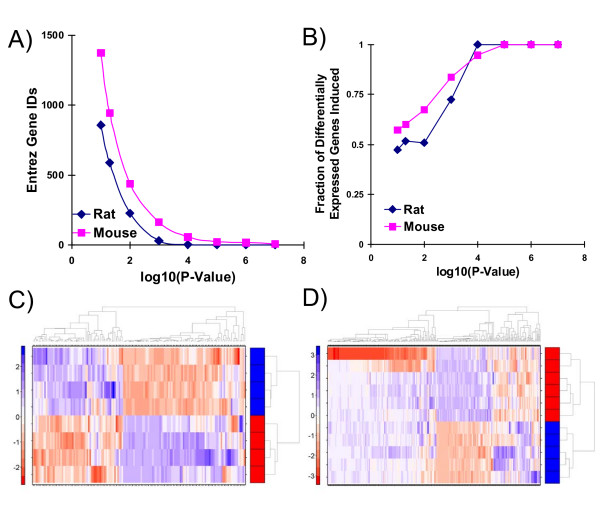
**TCDD-Induced Changes in mRNA in Rat and Mouse**. The hepatic mRNA abundance profiles of C57BL/6 mice and L-E rats were determined using microarray methods. Following GCRMA pre-processing, ProbeSet-wise linear-models were fit to identify differentially expressed genes. A) A plot of the number of distinct genes (Entrez Gene IDs) called differentially-expressed in each species (y-axis) as a function of threshold (x-axis) suggests more mouse genes than rat genes are TCDD-responsive. B) In a threshold-independent manner (x-axis) a larger fraction of mouse genes than rat genes are induced by TCDD. C) The variance of each rat ProbeSet was calculated and those having a variance above 1.0 were mean-centered and root-mean-square-scaled and subjected to divisive hierarchical clustering using the DIANA algorithm. TCDD-treated animals (rows with red annotation bars) cluster independently from vehicle controls (rows with blue annotation bars) in this unsupervised analysis. D) The variance of each mouse ProbeSet was calculated and those having a variance above 1.0 were mean-centered and root-mean-square-scaled and subjected to divisive hierarchical clustering using the DIANA algorithm. TCDD-treated animals (rows with red annotation bars) cluster independently from vehicle controls (rows with blue annotation bars) in this unsupervised analysis.

We first analyzed the rat and mouse data separately. The arrays from each species were pre-processed independently and subjected to ProbeSet-wise linear modeling. Following a multiple-testing correction, we identified extensive responses to TCDD in both species (Figure 1A). The MOE430-2 array used for the mouse studies contains 45,101 ProbeSets while the RAE230A array used for the rat experiments contains only 15,293. This difference may contribute to the larger apparent transcriptional response in mouse relative to rat (Figure 1A). Complete gene lists are available (Additional files 2 and 3).

The induction of phase-I enzymes in response to TCDD treatment is well-characterized [2] so we hypothesized that more genes would induced by exposure to TCDD than would be repressed. To test this we plotted the fraction of differentially-expressed genes that were induced as a function of the threshold (Figure 1B). At stringent p-value thresholds (p_adjusted _< 10^-3^) both species exhibited primarily inductive responses. At less stringent thresholds, however, the rat showed similar amounts of induction and repression whereas the mouse response remained predominately induction (Figure 1B).

Next, we selected those genes that are most variable in each species and subjected them to pattern-recognition analysis [32]. This unsupervised analysis perfectly separated treated from control animals for both rats (Figure 1C) and mice (Figure 1D), indicating that the largest trend in the dataset is the differential response to TCDD, not inter-individual variability.

### Conserved Transcriptional Responses to TCDD

To control for the different sizes and contents of the rat and mouse microarrays used in this study we performed an ortholog mapping. We used build 58 of the Homologene database to identify orthologous genes based on their Entrez Gene IDs. For each ortholog pair we selected a single representative ProbeSet. We tested four independent ProbeSet-selection techniques: maximum mean signal, maximum absolute fold-change, minimum p-value, and a Probe-to-Gene mapping that remapped all Probes on the array into ProbeSets corresponding to distinct Entrez Gene IDs [33]. Because all four methods yielded similar results (Additional file 4) all downstream analyses focused on ProbeSets aggregated using the minimum p-value method.

In total 8,125 orthologous genes were represented on both microarray platforms (Additional file 5). Of these only 33 exhibited statistically significant (p_adjusted _< 0.01) changes in response to TCDD in both species (Table 1). Further, 3 of these 33 genes (Ccbl1, Fmo1, and Tpm1) showed divergent responses – they were induced in one species and repressed in the other. The remaining 30 genes form a common "core" of species-independent responses to TCDD and include many of the best-characterized TCDD-inducible genes, such as Cyp1a1, Cyp1a2, Cyp1b1, Nqo1 and Tiparp. Some genes, despite being altered in both species, showed responses of divergent magnitude. For example, Inmt was repressed 2.3-fold in mouse liver, but 48.5-fold in rat liver. Pmm1 was induced 23.6-fold in mouse liver, but only 3.1-fold in rat liver. These divergences may reflect, in part, differences in the basal mRNA levels across species, but specialized microarray platforms are required to evaluate this [34].

**Table 1:** Responses to TCDD Common to Mouse and Rat

	**Entrez Gene ID**		**M Value**		**Gene Symbol**		**Mouse Gene Name**
		
**HID**	**Mouse**	**Rat**	**Mouse**	**Rat**	**Mouse**	**Rat**	
68062	13076	24296	9.58	11.06	Cyp1a1	Cyp1a1	cytochrome P450, family 1, subfamily a, polypeptide 1
68035	13078	25426	8.23	9.46	Cyp1b1	Cyp1b1	cytochrome P450, family 1, subfamily b, polypeptide 1
9167	99929	310467	6.83	4.07	Tiparp	Tiparp _predicted	TCDD-inducible poly(ADP-ribose) polymerase
695	18104	24314	4.52	3.05	Nqo1	Nqo1	NAD(P)H dehydrogenase, quinone 1
81752	21743	368066	-1.21	-5.66	Inmt	LOC368066	indolethylamine N-methyltransferase
90898	29858	300089	4.56	1.62	Pmm1	Pmm1	phosphomannomutase 1
38296	211446	252881	1.79	2.99	Exoc3	Exoc3	exocyst complex component 3
22419	12778	84348	1.36	3.19	Cxcr7	Cmkor1	chemokine (C-X-C motif) receptor 7
2412	18024	83619	2.35	2.15	Nfe2l2	Nfe2l2	nuclear factor, erythroid derived 2, like 2
56841	78798	313861	1.55	2.60	Eml4	Eml4 _predicted	echinoderm microtubule associated protein like 4
32722	76650	296271	1.90	2.10	Srxn1	Srxn1	sulfiredoxin 1 homolog (S. cerevisiae)
68082	13077	24297	2.36	1.27	Cyp1a2	Cyp1a2	cytochrome P450, family 1, subfamily a, polypeptide 2
2252	20969	25216	-1.16	-1.77	Sdc1	Sdc1	syndecan 1
31150	78610	308846	0.72	1.43	Uvrag	LOC308846	UV radiation resistance associated gene
31384	117198	289089	-0.68	-1.29	Ivns1abp	Ivns1abp_predicted	influenza virus NS1A binding protein
1952	207728	81743	1.10	0.84	Pde2a	Pde2a	phosphodiesterase 2A, cGMP-stimulated
375	18010	24591	0.45	1.27	Neu1	Neu1	neuraminidase 1
37872	70266	311844	-0.53	2.22	Ccbl1	Ccbl1	cysteine conjugate-beta lyase 1
41470	68371	171564	0.57	1.03	Pbld	Mawbp	phenazine biosynthesis-like protein domain containing
86978	28248	50572	-0.50	-1.07	Slco1a1	Slco1a1	solute carrier organic anion transporter family, member 1a1
55580	78943	498013	-0.69	-0.81	Ern1	RGD1559716 _predicted	Endoplasmic reticulum (ER) to nucleus signalling 1
134	14600	25235	-0.44	-1.05	Ghr	Ghr	growth hormone receptor
11098	64058	292949	0.37	0.97	Perp	Perp _predicted	PERP, TP53 apoptosis effector
4480	16796	29278	-0.75	-0.57	Lasp1	Lasp1	LIM and SH3 protein 1
11483	67819	362912	0.57	0.71	Derl1	RGD1311835	Der1-like domain family, member 1
55885	14661	24399	-0.27	-1.00	Glud1	Glud1	glutamate dehydrogenase 1
7673	12822	85251	-0.46	-0.52	Col18a1	Col18a1	procollagen, type XVIII, alpha 1
41475	109672	64001	0.55	0.41	Cyb5	Cyb5	cytochrome b-5
41077	66537	288455	0.56	0.32	Pomp	RGD1305831 _predicted	proteasome maturation protein
55884	11692	27100	0.47	0.36	Gfer	Gfer	growth factor, erv1 (S. cerevisiae)-like (augmenter of liver regeneration)
2090	19172	58854	0.29	0.38	Psmb4	Psmb4	proteasome (prosome, macropain) subunit, beta type 4
55520	14261	25256	1.13	-1.23	Fmo1	Fmo1	flavin containing monooxygenase 1
88552	22003	24851	1.09	-1.07	Tpm1	Tpm1	tropomyosin 1, alpha

Following pre-processing, a linear-modeling approach was used to identify ProbeSets associated with TCDD response in each species. ProbeSets were mapped to Entrez Gene IDs using the Affymetrix annotation (version na24). Orthologous genes were identified using the Homologene database (build 58). This table lists genes that display statistically significant (p_adjusted _< 0.01) changes in mRNA abundance in both species. The M-values represent the magnitude of difference in expression caused by TCDD exposure in log_2 _space. For example, Cyp1a1 has an M-value of 11.1 in rat, indicating 2,194-fold induction in response to TCDD treatment

**Figure 2:**
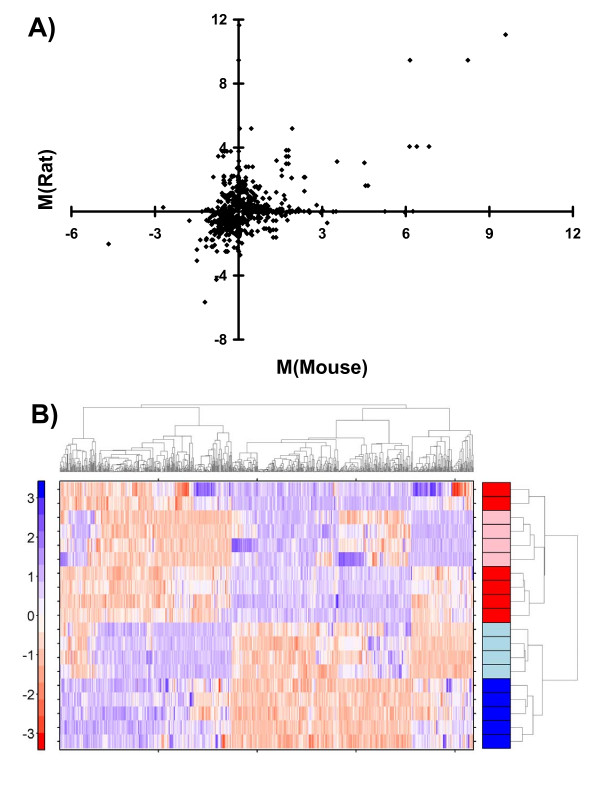
**Correlation of Rat and Mouse Responses to TCDD**. To assess the variability of the response to TCDD between mice and rats we took the pre-processed and linearly-modeled data and selected all ProbeSets with evidence for differential mRNA abundances (p_adjusted _< 0.01) in at least one species. We mapped homologs between the two species using the Homologene database. A) To determine if genes showed similar trends in their profiles we plotted the fold-change in log_2 _space (M-values) for all homologs. The two profiles are well-correlated (Spearman's rho = 0.32, p < 2.2 × 10^-16^), showing similar trends. B) To determine if these similarities were the strongest pattern within the dataset we selected all genes with evidence for TCDD-induced changes in mRNA abundance (p_adjusted _< 0.01 in one or both species) and selected the pre-processed values for all animals in both species. These values were then median-centered and root-mean-square-scaled separately within species before being subjected to divisive hierarchical clustering using the DIANA algorithm. The rat (pink) and mouse (red) TCDD animals cluster together, separately from the rat (light blue) and mouse (dark blue) controls. This unsupervised clustering suggests that species differences are less prominent than conserved TCDD-induced changes in mRNA abundances.

To assess if the direction of response to TCDD was conserved across species we plotted the fold-changes of the rat and mouse orthologs against one another (Figure 2A). The fold-changes are moderately but statistically significantly correlated (Spearman's rho = 0.32, p < 2.2 × 10^-16^), suggesting that orthologs respond similarly in the two species. This result is independent of the p_adjusted _threshold of 0.01 used (Additional file 1).

To corroborate this finding we again exploited machine-learning techniques. We extracted the GCRMA-pre-processed signal intensities for orthologous genes showing evidence for differential expression (p_adjusted _< 0.01) in either species. To normalize signal across species we employed gene-wise median-centering and root-mean-square-scaling. The normalized matrices were then merged gene-wise and subjected to divisive hierarchical clustering (Figure 2B). The six TCDD-treated mice (red) and four TCDD-treated rats (pink) cluster separately from the five control mice (dark blue) and four control rats (light blue). This demonstrates that a core response to TCDD is conserved across species. These results are independent of the significance threshold employed (Additional file 1). Scaling genes within each species is critical: if this normalization step is omitted (Additional file 1) or is performed after the two species are aggregated (Additional file 1) artefacts caused by the different microarrays used for each species predominate over the transcriptomic response.

### Divergent Transcriptional Responses to TCDD

Boverhof and coworkers found that 15.1% of rat genes and 12.8% of mouse genes that respond to TCDD show conserved responses [27]. Having constructed a list of 33 conserved responses to TCDD (Table 1), we asked if the low-level of conservation observed in the Boverhof et al. study would be replicated in a larger fraction of the transcriptome (8,125 orthologous genes in our study compared to 3,087 in the Boverhof analysis). Additionally, while we kept the mouse strain constant, we considered L-E rather than Sprague-Dawley rats: it has been established that different rat strains show large basal differences in their transcriptomes (Boutros PC et al., submitted).

**Figure 3:**
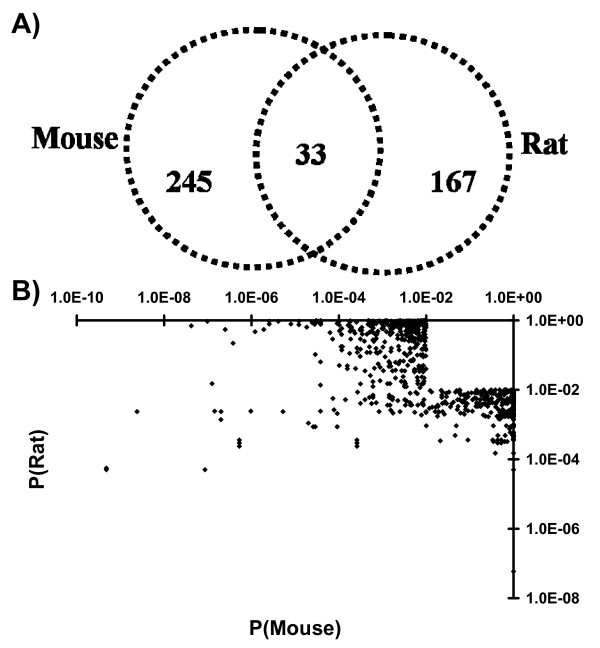
**Gene-Wise Differences in Rat and Mouse Responses to TCDD**. A) The specific identity of genes showing TCDD-induced changes in mRNA abundances diverges significantly between rats and mice. Only 33 genes are found altered in both species and, of these, 3 are altered in different directions. Thus only 15% of rat responses (30/200) and 10.8% of mouse responses (30/278) to TCDD are conserved in homologs from the other species. B) To test if genes with strong evidence (low p-values) for differential expression in one species would have similarly strong evidence (low p-values) in the other we plotted the adjusted p-values for the two species in log_10 _space. Surprisingly these values are strongly anti-correlated (Spearman's rho = -0.60, p < 2.2 × 10^-16^), suggesting that genes that have strong evidence for differential expression in one species are generally likely to have weak or no evidence in the other.

After surveying a large portion of the transcriptome we confirmed that only a fraction of TCDD-responses are conserved between mouse and rat (Figure 3A). Of the 278 genes that respond to TCDD in the mouse 10.8% are altered in the same direction in the rat. Of the 200 genes that respond to TCDD in the rat 15.0% are altered in the same direction in the mouse. Importantly these results are based on an analysis of only those 8,125 orthologous genes whose mRNA abundances were assayed in both species. These findings are independent of the p-value threshold selected (Additional file 1).

Given the weak correlation between changes in mRNA abundance in the two species (Figure 2A), we tested the possibility that the differences in gene-identities result from differences in statistical significance and thus represent artefacts of threshold selection. For example, genes that are TCDD-responsive in mouse might tend to show higher inter-animal variability in the rat, leading to the observed lack of overlap. We plotted the adjusted p-values for each gene on a log-scale (Figure 3B). No positive correlation was observed, disproving the hypothesis of threshold selection bias and suggesting that differences are caused by other factors. Indeed, a strongly negative correlation between p-values in the two species was calculated (Spearman's rho = -0.60, p < 2.2 × 10^-16^) indicating that genes that respond to TCDD in one species are less likely to respond in the other than predicted by chance alone. Again, this finding is independent of the p_adjusted _parameter selected (Additional file 1)

### Functional Analysis of TCDD-Responsive Genes

The above analyses identified large numbers of genes whose hepatic mRNA levels are altered by exposure to TCDD in a species-dependent manner, far more than are in a species-independent manner. Mice and rats exhibit mostly similar toxic outcomes to TCDD exposure, although they show some distinct morphological features in the liver (discussed further below). This could result from at least three mechanisms. First, aspects of hepatotoxicity common to both species may be mediated by the small "core" of species-independent responses. Second, toxicity might not be mediated by any of the mRNA changes in the liver. Third, toxicity might be mediated by different genes in each species, but these genes may ultimately lead to dysregulation of a common set of pathways, thereby leading to phenotypic similarities.

To test this third possibility, we studied the functional enrichment of genes that respond to TCDD in mice-only, in rats-only, or in both species using gene ontology analysis [35]. We identified the GO terms most significantly enriched in each group based on their false-discovery rate. Selected conditions are shown in Table 2 and the complete dataset is in Additional file 6. Several GO categories were strongly enriched in species-independent responders, including microsomal localization, electron transport, and unfolded protein response. One term, oxidoreductase activity, was enriched in all three groups. This indicates that a common core set of genes with oxidoreductase activity is dysregulated by TCDD in both species, with additional dysregulation of mouse- and rat-specific oxidoreductases. Finally, several species-specific pathways were identified, including mouse-specific dysregulation of translation (as previously identified [28]). Overall only one functional group met our initial criteria of showing regulation in both species through different gene sets – lipid metabolic processes.

**Table 2:** Selected Enriched Gene Ontology Categories

**GO ID**	**Rat**	**Mouse**	**Common**	**GO Term**
GO:0006118	***3.33E-04***	3.43E-01	***8.43E-03***	electron transport
GO:0006984	1.01E+00	1.00E+00	***9.67E-03***	ER-nuclear signaling pathway
GO:0006986	1.02E+00	1.01E+00	***1.10E-02***	response to unfolded protein
GO:0005792	2.89E-01	7.64E-01	***1.30E-02***	microsome
GO:0020037	7.99E-01	9.21E-01	***2.61E-02***	heme binding
GO:0016491	***1.00E-04***	***2.71E-03***	***4.02E-02***	oxidoreductase activity
GO:0005832	9.99E-01	***2.82E-02***	1.01E+00	chaperonin-containing T-complex
GO:0006412	1.04E+00	***1.11E-04***	1.02E+00	translation
GO:0030529	1.04E+00	***1.00E-04***	1.02E+00	ribonucleoprotein complex
GO:0030151	1.01E+00	***1.81E-02***	1.03E+00	molybdenum ion binding
GO:0008610	***7.20E-02***	5.74E-01	1.05E+00	lipid biosynthetic process
GO:0044255	***1.18E-02***	***3.72E-02***	1.15E+00	cellular lipid metabolic process

Genes differentially expressed in each species were selected at p_adjusted _< 0.01 using linear-modeling analysis. The set of genes whose mRNA levels were altered by TCDD in only mice, only rats, or in both species were separately subjected to Gene Ontology enrichment analysis to identify specific pathways or functions modulated. A selection of enriched GO terms is shown. The numeric values are false-discovery rates estimated by 1,000 permutations of the input data. Only a subset of GO categories is shown. Bold text indicates those categories significant at a 10% false-discovery rate.

### Genomic Localization of TCDD-Responsive Genes

**Figure 4:**
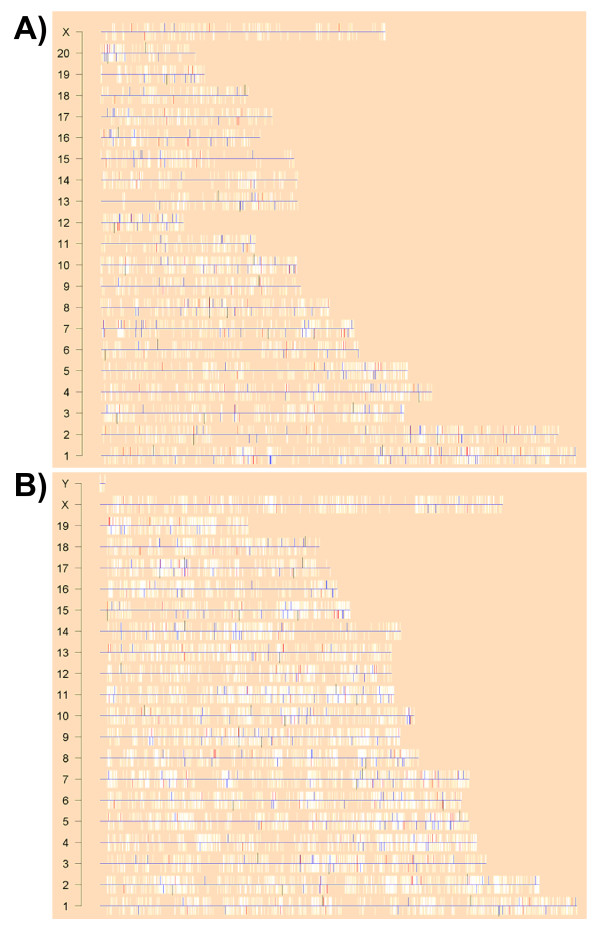
**Genome-Wide Mapping of Differential Expression**. To determine if differentially-expressed ProbeSets were localized to specific portions of the rat or mouse genome we plotted the entire genome, with one chromosome per line. Each gene was plotted with a white bar representing its location on the chromosome and its position on the plus (up) or minus (down) strand. Genes showing differential abundances (p_adjusted _< 0.01) in the rat are colour-coded in red, those in the mouse in blue, and those in both species in black. No prominent clusters of expression are observed in either the rat (A) or mouse (B).

Having demonstrated large-scale differences between the rat and mouse transcriptional responses to TCDD and having shown that these differences lead to alterations in specific pathways, we next considered mechanism. One obvious mechanism for differences in mRNA expression is changes in gene dosage. Indeed many mRNA expression studies, including those of untreated rat liver, have found localized domains or "islands" of differential expression (Boutros PC et al., submitted). We assessed the genomic localization of species-specific and species-independent responses to TCDD in both species. No large regions or islands are apparent amongst the common (black), the rat-specific (red), or the mouse-specific genes (blue) in either the rat (Figure 4A) or the mouse (Figure 4B) genomes.

### Transcriptional Regulation of TCDD-Responsive Genes

Another mechanism that might underlie differences in the transcriptional response to TCDD between mouse and rat is the differential activity of one or more regulatory genes. Alterations in the activities of a small number of regulatory genes, such as transcription factors, could lead to the large number of mRNA differences observed between species. One way to find evidence for differential transcription factor activities is to use transcription-factor binding-site (TFBS) enrichment analysis [36]. We searched the promoter regions of rat-specific, mouse-specific and common-responders for enrichment using the JASPAR library of TFBSs [37]. These searches were performed using both the rat and mouse promoters in an effort to understand species-differences in promoter architecture.

Sixty separate TFBS motifs were enriched in at least one of the six datasets searched (Additional file 7). For example, the p53 binding-site is enriched in the mouse promoter regions of all TCDD-responsive genes, regardless of whether or not those genes responded in the rat. Surprisingly neither the three AHRE-I variants nor the AHRE-II motif exhibited enrichment in any of the six datasets. Instead the anomalous Cyp1b1 complex identified in mouse was found to be enriched in the promoters of the mouse orthologs of rat-specific genes [38]. Visual inspection did not reveal significant similarity between the mouse and rat orthologs; indeed in some cases they showed divergent trends: species-independent genes were enriched for the Nr2f1 (COUP-TF1) binding-site in mouse, but depleted for it in rat. Only one motif, Fos, was enriched in the species-independent genes in both rat and mouse but not in any of the other datasets.

**Figure 5:**
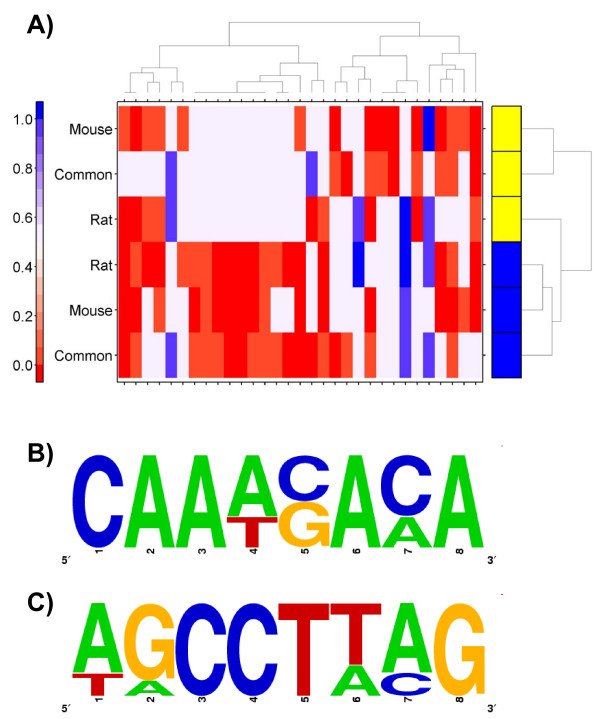
**Analysis of Transcription-Factor Binding-Sites**. To rationalize the observed patterns of species-dependent and species-independent expression we performed two separate transcription-factor binding-site analyses. A) We employed a library-based position-weight matrix enrichment analysis using the JASPAR library and the clover algorithm. We separately tested rat (rows with yellow boxes) and mouse (rows with blue boxes) promoter sequences from rat-specific, mouse-specific, and common genes. Thus the first row indicates the analysis of the promoter sequences of the rat orthologs of genes displaying mouse-specific TCDD expression. Each column represents a separate position-weight matrix, and only matrices enriched or depleted in at least two datasets are included. The colour bar indicates the p-value based on 10,000 randomizations. B) and C) We also performed *de novo *motif discovery using the MotifSampler algorithm. Two matrices enriched in genes showing species-independent responses to TCDD were converted into sequence logos and are displayed here. Neither sequence appears to match a known transcription-factor binding-site.

To help visualize and rationalize this divergence we employed unsupervised machine-learning. We selected all motifs that were enriched or depleted in at least two of the six datasets and set the p-values to 0.5 for all not-significant values. When the resulting matrix was clustered (Figure 5A) a clear separation between the mouse and rat promoters was revealed. Rather than clustering according to TCDD-responsiveness, the data clustered according to the species of origin. This strongly suggests that species-specific regulatory differences predominate, in agreement with the divergent transcriptional profiles observed.

While TFBS enrichment analyses can rationalize expression profiles, they are limited by the size and scope of the underlying TFBS library. Because only a small fraction of transcription factors have been analyzed in sufficient detail to have a position-weight matrix generated, a large fraction of regulatory genes are necessarily omitted. To complement this approach we thus chose to use a second technique: pattern-discovery analyses.

Pattern discovery algorithms search for recurrent motifs within sequences. To identify novel sequence motifs associated with species-dependent and species-independent responses to TCDD we employed the well-established MotifSampler algorithm [39] to identify novel motifs associated with each group of genes. This analysis identified a series of novel motifs putatively associated with the response to TCDD (Additional file 8). Two of these motifs, derived from the promoters of species-independent genes, are shown as sequence logos in Figure 5B. Neither of these motifs appears to match the affinities of any currently known transcription-factor.

### Comparison With Previous Study by Boverhof et al

**Figure 6:**
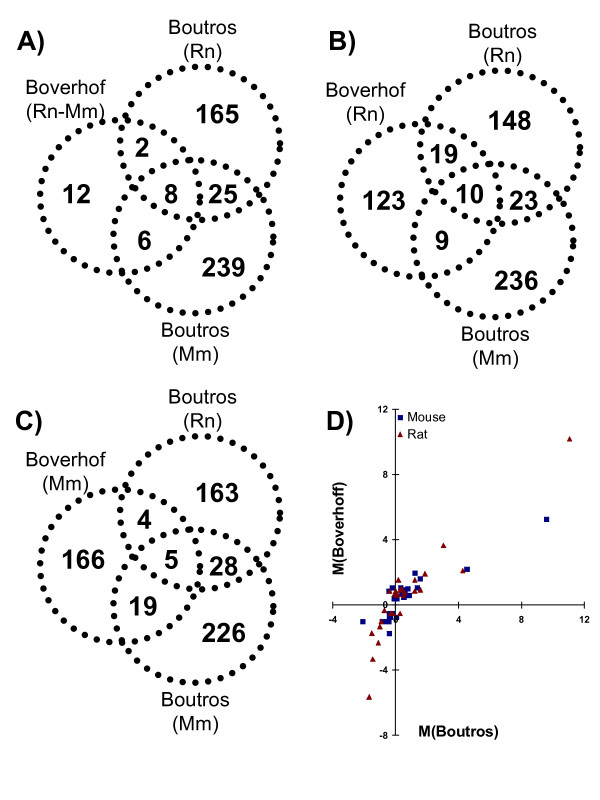
**Comparison With Previously Published Data**. We compared our findings with those of Boverhof and co-workers by performing a gene-by-gene comparison. Responsive genes were identified in our dataset as those having p_adjusted _< 0.05. A) First, we looked at the 32 genes that responded to TCDD in both species in the Boverhof et al. study and assessed their response in our dataset. Four genes could not be mapped to homologs on arrays, while 57% (16/28) genes responded to TCDD in one or two species in our study. We then repeated this analysis looking at the rat-specific (B) and mouse-specific (C) genes identified in the Boverhof study. In total 24 of the 185 rat-specific genes and 31 of the 225 mouse-specific genes could not be mapped to the 8,125 ortholog pairs present on our oligonucleotide arrays. Next we mapped the specific fold-changes observed in the two studies for both species (D). Highly similar patterns were observed for both mouse (Spearman's rho = 0.83, p = 4.4 × 10^-8^) and rat (Spearman's rho = 0.88, p = 1.06 × 10^-9^).

The minimal overlap in responses to TCDD between mouse and rat described here recapitulates and extends previous work [27]. To directly compare the specific genes detected in the two studies, we performed a gene-by-gene comparison. We first focused on the 32 genes identified by Boverhof and coworkers as responding to TCDD in both species and mapped them to our dataset using mouse Entrez Gene IDs (Figure 6A). Four of the 32 genes could not be mapped. We assessed the TCDD response of the remaining 28 genes in our dataset: 8 were TCDD responsive in both species, 8 were TCDD responsive in one species, and 12 were unresponsive. In total, 57% (16/28) of the genes previously identified by Boverhof et al. responded to TCDD in our dataset in at least one species.

We next replicated this analysis by looking at the list of rat-specific (Figure 6B) and mouse-specific (Figure 6C) genes identified in the Boverhof study. In each of the species-specific datasets we see greater overlap between the Boverhof findings and those reported here for the same study. For example, 225 genes were identified as responding to TCDD in mice only in the Boverhof study. Of these, 19 responded in our mouse analysis but only 4 in our rat analysis. Notably, more genes showed TCDD responsiveness in only one of the two studies than did in both studies.

Reasoning that many differences between the two studies might reflect the different statistical methodologies and numbers of animals, we next compared the magnitude of TCDD responses for the 28 mapped genes that showed species-independent responses (Figure 6D). The resulting plots are highly correlated for both mouse and rat, indicating that indeed the two array studies generated highly similar results.

## Discussion and conclusion

The goal of this study was to compare and contrast the transcriptional response to TCDD in two closely-related rodent species, the rat and the mouse. Because these are the two most commonly used model organisms in pharmacology and toxicology, large divergences in their response to particular compounds is of broad interest. Previous work using cDNA microarrays containing 3,087 orthologous genes found that, while hundreds of genes responded to TCDD, only a small core of 10–20% was common to both species [27]. We sought to evaluate and extend these findings.

We exploited oligonucleotide arrays that contain 8,125 ortholog pairs – 2.6-fold more than previously studied. We used a single dose, chosen to represent approximately equivalent multiples of the individual species' LD_50 _values. We assessed expression after 19 hours of TCDD exposure. The selected dose should be sufficient to eventually induce all major overt toxicities of TCDD and the selected time-point should be early enough to allow us to focus on primary responses rather than on the secondary manifestations of toxicity. We further broadened the mouse/rat comparison by using the highly TCDD-sensitive L-E strain, whereas the Boverhof study analyzed Sprague-Dawley rats.

We found that responses to TCDD are moderately correlated between the two species (Figure 2A), suggesting a broad trend towards similar responses. Further, a novel bi-species clustering approach shows in an unsupervised and unbiased way that the strongest signal within the dataset is the response to TCDD, not the difference between species (Figure 2B). We also show that hundreds of genes respond to TCDD in each species (Figure 1A), but only a small core of 10–20% was common to both – corroborating the findings of Boverhof and coworkers (Figure 3A).

This core set of species-independent TCDD-responsive genes contains many well-characterized genes, including Cyp1a1, Cyp1a2, Cyp1b1, Nqo1, and Tiparp – all of which have been previously detected in high-throughput studies [27,28,40]. However, several species-independent genes have not previously been well-characterized for their response to TCDD but have functions that may be related to the clearance of xenobiotics. For example: indolethylamine N-methyltransferase is an enzyme that methylates indoles such as tryptamine, tagging them for degradation; cysteine conjugate-beta lyase metabolizes cysteine conjugates of alkanes and alkenes. Gene ontology analysis shows that the species-independent genes are enriched for microsomal genes, genes involved in the unfolded protein response, and genes involved in ER-nuclear signalling.

The 30 genes showing concordant responses to TCDD in rat and mouse represent prime candidates for explaining TCDD-induced hepatotoxicities. However, these toxicities might result in at least three other ways. First, toxicities may arise from events outside the liver and thus be independent of changes in hepatic mRNA levels. Indeed, it has been shown that hematopoietic cells contribute to the hepatic lesions caused by TCDD [41]. Second, if toxicities arise from a cell-type present at a low frequency in the liver rather than from hepatocytes, relevant mRNA changes may be below the threshold of detection of our platform. For example, the Kupffer cells appear to play a central role in the hepatic response to many toxic and carcinogenic agents [42] and the Ito (stellate) cells are responsible for retinoid storage and metabolism in liver [43], a target for TCDD action [44]. Third, toxicities may arise by perturbation of common pathway through different genes in each species.

Testing the first two hypotheses will require the use of tissue-specific transgenic animals and are thus beyond the scope of this study. To test the idea that toxicity results from the dysregulation of a pathway common to mouse and rat we used gene-ontology enrichment analysis. We searched for dysregulated functional groups in the lists of rat-specific and mouse-specific genes and found only one pathway dysregulated in both species – cellular lipid metabolism. Because this minimal overlap could occur by chance alone, and with caveats regarding tissue-specificity and transcriptome-coverage, it appears likely that the genes mediating TCDD-induced toxicity are amongst those reported in Table 1. While these species-independent genes appear skewed towards up-regulation, it is not clear that highly up-regulated genes are mediators of hepatic toxicities. For example, CYP1A1 and CYP1A2 are both highly induced in the liver of TCDD-resistant Han/Wistar (*Kuopio*) rats [25]. CYP1A1 can modulate some of the hepatotoxic effects of TCDD, as shown by lack of hepatocyte hypertrophy and uroporphyria in *Cyp1a1*^-/- ^mice [45], but induction of CYP1A enzymes is not, in itself, sufficient to cause hepatotoxicity.

It would be of interest to repeat our analysis using expression profiles collected 96 to 240 hours after TCDD exposure, when overt toxicities are manifest. At these later time-points it would no longer be possible to discern primary from secondary events, but instead there would be an increased ability to contrast the final toxic pathways perturbed in each species.

While about 15% of TCDD-responsive genes are species-independent and thus potentially mediate TCDD toxicities, the roles of the remaining 85% of genes are intriguing to consider. There are two key questions. First, does altered expression of these genes have functional consequences? Second, what mechanisms mediate species-specific responses?

If the species-independent genes had no functional consequence we might expect them to be randomly distributed across different functional classes. Instead, gene-ontology enrichment analysis (Table 2) shows that they represent specific responses. In particular, the formation and function of ribonucleoprotein complexes involved in translation is significantly dysregulated in mice but not in rats. In our study of AHR-knockout mice we showed that this effect is AHR-dependent [28] and suggested that it may mediate some TCDD-toxicities. Because this effect is absent in the rat, this pathway may contribute to degenerative changes induced by TCDD that are more prominent in mouse liver relative to rat liver.

The vast majority of TCDD toxicities are common to both species, but some species-specific differences do exist. For example, multinucleated giant hepatocytes occur only in rats, while hydropic degeneration, fat accumulation, inflammatory cell infiltration as well as apoptosis and necrosis are more pronounced in mice [27,40,41,46-48]. We selected our TCDD dose to reflect approximately equal multiples of the LD_50 _values for each species to ensure that a similar ultimate phenotype was being considered. Nevertheless it is possible that the type of hepatic injury is species-specific and that this leads to the observed differences in transcript profiles. Several lines of evidence suggest against this possibility, including the evolutionary proximity of rat and mouse, the similar responses of known TCDD-responsive genes, and the inter-species similarity of the vast majority of morphological changes. Further work will be needed to clarify between these two situations.

Many different mechanisms can be proposed to explain the large species-specific responses observed, including copy-number variation, differences in transcription-factor binding affinities, and differential transcription-factor activities. We did not find evidence that copy-number variation plays a role (Figure 4), so we focused on the potential role of transcription-factors in species-specific responses.

It has been established that transcription-factor binding is poorly correlated with changes in mRNA expression [49]. Further, when the transcriptional regulatory networks were compared between human and mouse liver, they were found to differ dramatically [50]. To determine whether specific transcription-factors are associated with the species-specific responses we performed two separate analyses of transcription-factor binding-site motifs. First we used a library-based enrichment analysis [51] with a database of 130 known motifs that we supplemented with 5 AHR-associated motifs [37]. Because the majority of sequence-specific DNA-binding proteins do not have known binding-site motifs, we complemented this analysis with an unsupervised analysis using the MotifSampler pattern-discovery algorithm [39].

Our library-based analysis identified numerous motifs enriched in both species-dependent and species-independent manners, some clearly associated to the biology of dioxin-toxicity. For example, the enrichment of p53 motifs in mouse promoters suggests that a p53-dependent apoptotic response was initiated in mouse, but not in rat. Apoptosis is a common sequela to TCDD exposure in mouse liver [41,47] and, while it also occurs in rats, it appears less frequent [52]. One motif, Nr2f1 (COUP-TF1) showed divergent enrichment between the promoters of mouse and rat that responded similarly to TCDD. The AHR has been shown to directly bind COUP-TF1 in human cells [53]. When we consider the overall patterns of TFBS-enrichment in an unbiased manner using unsupervised machine-learning, however, we found that species-specificity was the dominant trend. This suggests that the transcriptional regulatory networks are sufficiently diverged in mouse and rat as to make non-phylogenetic motif analysis highly challenging [50].

MotifSampler is a variant of Gibbs sampling that introduces a higher-order Markov background model and incorporates a Bayesian mechanism to estimate the missing value: the number of motifs occurring in each sequence [39]. MotifSampler was applied here to six different promoter datasets to successfully identify novel motifs. Biochemical analyses are required to demonstrate the functionality of these motifs, especially in the absence of an appropriate validation dataset, but the success of this analysis suggests that significant components of the AHR transcriptional network remain to be elucidated.

We directly compared our results to those of Boverhof and coworkers at the gene- level (Figure 6). This is important because the two studies differed in animal handling, time-points, strain of rat used, gender and age of animals, array platforms employed, and statistical methodologies. Despite all these differences Spearman's correlations above 0.8 were observed between the two studies for both mouse and rat – an outstanding concordance. However, despite this correlation it is notable that the majority of genes identified as TCDD-responsive were found in only one of the two studies. This provides a cautionary note to the use of toxicogenomics for high-throughput testing, and stands in contrast to the optimistic findings of the MAQC study [54,55].

Thus we have shown that most TCDD-responsive genes are species-specific and that the small core of species-independent genes shares both common functions and common transcriptional-regulatory elements. Taken together these data suggest that the vast majority of dioxin-responsive genes play no direct role in the toxic responses. Rather, it may be that a small number of regulatory genes under direct AHR control lead to dramatic inter-species differences. It would be highly advantageous to further refine the list of genes associated with dioxin-toxicities by profiling the transcriptional response of additional strains of mouse and rat, or by exploiting additional rodent species.

This study has significant implications for the use of rodent models to understand mechanisms of pharmacological or toxicological compounds. If transcriptional responses have been extensively remodelled in rat and mouse since their last common ancestor then hepatic expression studies may yield contradictory and species-specific results. It may be necessary to test both species and focus on the subset of overlapping genes. An optimal study design might include several strains of mice and several strains of rat, thereby focusing the list of transcriptional responses to a small number that exhibit both intra- and inter-species homogeneity and are thus highly likely to be directly involved in the phenotypic effects of the compound under investigation.

## Methods

### Animal Handling

#### Rats

The Long-Evans (*Turku/AB*) (LE) strain of rat was selected for these analyses because it harbours a wild-type Aryl Hydrocarbon Receptor (AHR) and is the most TCDD-sensitive rat strain reported, with an acute LD_50 _of 9.6 (females) or 17.7 (males) μg/kg [1]. Male animals, 10–12 weeks of age, were grown in breeding colonies of the National Public Health Institute, Division of Environmental Health, Kuopio, Finland. All animals were males 10–12 weeks old. They were housed in groups of 4 rats (an entire treatment group per cage) in suspended stainless-steel wire-mesh cages with pelleted R36 feed (Lactamin, Stockholm, Sweden) and tap water available *ad libitum*. The temperature in the animal room was 21 ± 1°C, relative humidity 50 ± 10%, and lighting cycle 12/12 hours light/dark. The study plans were approved by the Animal Experiment Committee of the University of Kuopio and the Kuopio Provincial Government. Liver was harvested between 8:30 and 11:00 from rats treated by gavage with a single 100 μg/kg dose of TCDD or with corn oil vehicle for 19 hours. Four control (corn-oil treated) and four experimental (TCDD-treated) rats were used in this study.

#### Mice

Wildtype (Ahr^+/+^) C57BL/6 mice harbouring wild-type AHR alleles were bred at the National Public Health Institute, Kuopio, Finland from stock originally obtained from The Jackson Laboratory. At 15 weeks of age mice were given a single dose of 1000 μg/kg TCDD or corn oil vehicle by gavage. TCDD initially was dissolved in ether and added to corn oil; the ether subsequently was evaporated off. Liver was harvested 19 hours after treatment, sliced, snap-frozen and stored in liquid nitrogen until homogenization. Five control (corn-oil treated) and six experimental (TCDD-treated) mice were used in this study.

TCDD for both experiments was purchased from the UFA-Oil Institute (Ufa, Russia) and was >99% pure as determined by gas chromatography-mass spectrometry.

Total RNA was extracted from both rat and mouse livers using Qiagen RNeasy kits according to the manufacturer's instructions (Qiagen, Mississauga, Canada). Total RNA yield was quantified by UV spectrophotometry and RNA integrity was verified using an Agilent 2100 Bioanalyzer (Agilent Technologies, Santa Clara, CA).

### Pre-Processing and Statistical Analysis of Microarray Data

Affymetrix RAE230A and MOE430-2 arrays were run according to manufacturer's protocols at The Centre for Applied Genomics at the Hospital for Sick Children (Toronto, Canada). Four independent biological replicates (individual rats) were analyzed for each condition (8 total arrays), while five control and six treated mice were analyzed (11 total arrays). The raw array data have been deposited into the GEO repository with accessions GSE10769 (mouse) and GSE10770 (rat).

Microarray data were loaded into the R statistical environment (v2.6.2) using the affy package (v1.16.0) [56]. These data were pre-processed using the GC-RMA version of the RMA pre-processing algorithm [57], as implemented in the gcrma package (v2.10.0). Data were investigated for spatial and distributional homogeneity. The mouse data are part of a study of AHR^-/- ^animals described elsewhere [28], but were pre-processed separately for this study to avoid introducing bias by including AHR^-/- ^mice in the quantile-normalization step of pre-processing. The data for control rats have been analyzed separately elsewhere (Boutros et al. submitted), while the data from TCDD-treated rats have not. Again, pre-processing was performed separately for this study to avoid introducing bias.

All clustering analyses employed divisive hierarchical clustering using the DIANA algorithm as implemented in the cluster package (v1.11.10) and with Pearson's correlation as a similarity metric. Heatmaps were visualized using the lattice (v0.17-6) and latticeExtra (v0.3-1) packages. Subsets for clustering were selected using global variance thresholds. When both species were clustered together, scaling was performed separately for the mouse and the rat arrays to ensure that probe, normalization or loading differences did not bias the analysis.

### ProbeSet Annotation and Homolog Identification

To identify TCDD-induced changes in mRNA abundance model-based t-tests were fit using the limma software package (v2.12.0) and subjected to an empirical Bayes moderation of the standard error [58]. P-values from this analysis were corrected for multiple testing with a false-discovery rate adjustment [59]. We set our significance threshold at p_adjusted _< 0.01 for most downstream analyses. At this level we would expect no more than 1% of our hits to be false-positives. We also considered the p-value sensitivity of the overall analysis by varying the threshold from 10^-1 ^to 10^-7 ^in log steps and calculating the number of differentially-expressed ProbeSets at each threshold.

Genes were annotated with version na24 of the Affymetrix NetAffx annotation . Genomic localization of TCDD-responsive genes was performed using the geneplotter (v1.16.0), annotate (v1.16.1), mouse4302 (v2.0.1) and rae230a (v2.0.1) packages, all in version 2.6.2 of the R statistical environment.

Homologs were mapped between the two arrays using the Homologene database (build 58). Genes were mapped according to Entrez Gene ID to Homologene IDs and only genes present in both species were retained for downstream analyses. When multiple ProbeSets were present for a single Entrez Gene ID they were collapsed by selecting the ProbeSet with the minimum p-value. We compared this approach to selecting the ProbeSet with the maximum coefficient or the one with the maximum average signal, and only minor differences were observed amongst the three strategies. We also contrasted these three approaches with a sequence-based annotations provided by the custom CDF annotations mapping individual Probes to Entrez Gene IDs, as provided in BioConductor (v1.9) [33].

### Transcription-Factor Binding-Site Analysis

We used a library-based method to search for transcription-factor binding-sites enriched or depleted in specific gene sets [36]. Using the CLOVER software package [51] we queried a 2005 version of the JASPAR database containing 130 position-weight matrices [37]. We supplemented the JASPAR matrix with five AHRE-associated motifs as previously tested using phylogenetic footprinting [60]. To ensure that our results were robust, three separate permutation tests were used. We employed simple mononucleotide and dinucleotide randomization. Additionally, a background dataset containing the promoters of all 8,125 genes present on both arrays was generated and used. For each permutation test 10,000 randomizations were performed with a p-value threshold of 0.05 and a score threshold of 5. Only motifs significantly enriched or depleted in all three tests are reported. Genomic sequences from -1,000 to +1,000 relative to the transcriptional-start site were used, for sequences with a total length of 2,001 bp. These sequences were extracted from build rn4 of the rat genome and build mm8 of the mouse genome using annotation from the UCSC genome browser database downloaded on 2007-04-07 [61]. We separately tested the promoters in both species for motif enrichment in genes common to both species, genes specific to mouse, and genes specific to rat, leading to six total comparisons.

These same comparisons were also made using a pattern-discovery algorithm, MotifSampler [39]. First, the promoter regions for all orthologous pairs included in our expression array analyses were extracted from the genome for each species, as described above. Next, a third-order background model was generated for each species from all promoter regions by using the background-generator program provided with MotifSampler. Next, each dataset was analyzed at three pattern-lengths – 6, 8, and 10 bp – and at each length the analysis was repeated twice with the five highest scoring motifs retained from each run. Thus we analyzed three datasets, each in two species, and each dataset was analyzed at three pattern lengths. For each pattern-length ten motifs were generated, leading to a total of 180 novel motifs generated in this pattern-discovery analysis.

Unsupervised machine-learning was performed as described above, using divisive hierarchical clustering through the DIANA algorithm as implemented in the cluster package (v1.11.10) and with Pearson's correlation as a similarity metric. Heatmaps were visualized using the lattice (v0.17-6) and latticeExtra (v0.3-1) packages.

### Gene Ontology Enrichment analysis

Functional enrichment analysis was performed using the GOMiner tool [35]. Analysis was done using all mouse databases, look-up options, ontologies, and evidence levels. False-discovery rates were estimated with 1,000 randomizations and a 10% FDR threshold was set.

## Authors' contributions

PCB performed most bioinformatic and all statistical analyses and wrote the first draft of the paper. RY performed the motif-discovery analyses. ABO and RP guided the study design. All authors contributed to the study design and provided input into the final manuscript.

## Supplementary Material

Additional File 1 Figures demonstrating supplementary findings. Demonstrations of array quality, parameter insensitivity of analyses, and the importance of appropriate scaling in cross-species clustering. Click here for file

Additional File 2 Microarray Data for All Mouse ProbeSets. Annotation and linear-modeling fits for all ProbeSets on the MOE430-2 array. Click here for file

Additional File 3 Microarray Data for All Rat ProbeSets. Annotation and linear-modeling fits for all ProbeSets on the RAE230A array. Click here for file

Additional File 4 Homolog Response Analysis. Comparison of four separate methods of mapping homologs between rat and mouse. Click here for file

Additional File 5 Microarray Data for All Ortholog Pairs. Annotation and linear-model fits for ortholog pairs significantly affected by TCDD in both rat and mouse. Click here for file

Additional File 6 Gene Ontology Analysis. Complete results of gene ontology analyses. Click here for file

Additional File 7 Motif Enrichment Analysis Using CLOVER. Complete results of promoter analysis using the CLOVER program and the JASPAR library. Click here for file

Additional File 8 *de novo *Motif Discovery Using MotifSampler. Top hits from MotifSample *de novo *motif discovery analysis. Click here for file
