# Supplementary material for: Transcriptomic responses to 2,3,7,8-tetrachlorodibenzo-p-dioxin (TCDD) in liver: Comparison of rat and mouse
Source: BMC Genomics. 2008 Sep 16;9:419. doi: 10.1186/1471-2164-9-419 (PMC2559853; doi:10.1186/1471-2164-9-419)
Supplement: Additional File 1 — Figures demonstrating supplementary findings. Demonstrations of array quality, parameter insensitivity of analyses, and the importance of appropriate scaling in cross-species clustering. [file 1471-2164-9-419-S1.pdf]

Supplementary Figure 1: Quality Assessment of Mouse Arrays

The mouse microarray data were subjected to an extensive quality-control procedure prior to conducting downstream analyses. We assessed the spread in intensity distributions before (A) and after (B) GCRMA-pre-processing. We verified that each sample displayed similar RNA degradation characteristics, as indicated by having similar slopes in a plot of Probe intensity vs. position along the transcription (C). Finally we showed that biological replicates clustered together when using the unnormalized data.

Supplementary Figure 2: Quality Assessment of Rat Arrays

The rat microarray data were subjected to an extensive quality-control procedure prior to conducting downstream analyses. We assessed the spread in intensity distributions before (A) and after (B) GCRMA-pre-processing. We verified that each sample displayed similar RNA degradation characteristics, as indicated by having similar slopes in a plot of Probe intensity vs. position along the transcription (C). Finally we showed that biological replicates clustered together when using the unnormalized data.

Supplementary Figure 3: Parameter Sensitivity of M-Value Correlation Analysis

To assess the variability of the response to TCDD between mice and rats we took the pre-processed and linearly-modeled data and selected all ProbeSets with evidence for differential mRNA abundances in at least one species. We mapped homologs between the two species using the Homologene database. To determine if genes showed similar trends in their profiles we plotted the fold-change in $\log_2$ space (M-values) for all homologs. A) At $p_{adjusted} < 0.05$ the two profiles are correlated (Spearman's rho = 0.28, $p < 2.2 \times 10^{-16}$), showing similar trends in direction. B) At $p_{adjusted} < 0.001$ the two profiles

are also correlated (Spearman's rho = 0.31, $p = 6.12 \times 10^{-6}$), showing that this finding is independent of the p-value threshold selected.

Supplementary Figure 4: Parameter Sensitivity of Cross-Species Clustering

Some microarray analyses have been described to be sensitive to the selection of specific statistical parameters. For example gene-ontology enrichments may only occur if a gene-list is thresholded at $p < 0.01$ but not $p < 0.05$ or $p < 0.001$. Because threshold selection is ultimately arbitrary, it is important that major conclusions are independent of parameter choices. We demonstrated that mouse and rat orthologs cluster according to TCDD-treatment status (Figure 2B) by selecting genes that were statistically significant at $p_{adjusted} < 0.01$. To verify parameter-independence we selected genes at $p_{adjusted} < 0.05$ (A) and $p_{adjusted} < 0.001$ (B) and subjected them to the same median-centering, root-mean-square-scaling, and divisive hierarchical clustering as was done previously. In each case the mouse (red) and rat (pink) TCDD-treated animals cluster separately from the mouse (dark blue) and rat (light blue) control animals. Clustering of the genes dimension (B) shows that common-responsive genes (yellow) group together and are few in number relative to rat-specific (red) and mouse-specific (blue) responders.

Supplementary Figure 5: Cross-Species Clustering With Unscaled Data

The use of appropriately normalized data is critical for higher-order statistical analyses. For example, when we co-clustered rat and mouse data (Figure 2B) the data were scaled within species prior to executing the pattern-recognition analysis. This step was necessary because the individual ProbeSet-intensities for a given gene can vary between species as a result of the region of the gene targeted, the presence of cross-hybridizing transcripts, and the use of different quantile-normalization targets. We explored the effect of omitting this step from the analysis using sets of genes selected at $p_{adjusted} < 0.05$ (A), $p_{adjusted} < 0.01$ (B), and $p_{adjusted} < 0.001$ (C). In each case the TCDD-

treated (pink) and control (light blue) rats clustered together, separate from the TCDD-treated (red) and control (blue) mice. Clustering of the genes dimension (C) shows that common-responsive genes (yellow) group together and are few in number relative to rat-specific (red) and mouse-specific (blue) responders.

Supplementary Figure 6: Cross-Species Clustering With Global Scaling

The use of appropriately normalized data is critical for higher-order statistical analyses. For example, when we co-clustered rat and mouse data (Figure 2B) the data were scaled within species prior to executing the pattern-recognition analysis. This step was necessary because the individual ProbeSet-intensities for a given gene can vary between species as a result of the region of the gene targeted, the presence of cross-hybridizing transcripts, and the use of different quantile-normalization targets. We explored the effect of omitting this step from the analysis (Supplementary Figure 5), and found that removing it eliminated our ability to determine toxicological effects. Next we explored the effect of using a global, per-gene scaling procedure across all animals, rather than by species. Tets of genes were selected at $p_{adjusted} < 0.05$ (A), $p_{adjusted} < 0.01$ (B), and $p_{adjusted} < 0.001$ (C) and subjected to divisive hierarchical clustering, as before. In each case the TCDD-treated (pink) and control (light blue) rats clustered together, separate from the TCDD-treated (red) and control (blue) mice. Clustering of the genes dimension (C) shows that common-responsive genes (yellow) group together and are few in number relative to rat-specific (red) and mouse-specific (blue) responders.

Supplementary Figure 7: Parameter Sensitivity of Overlap Analysis

Some microarray analyses have been described to be sensitive to the selection of specific statistical parameters. For example gene-ontology enrichments may only occur if a gene-list is thresholded at $p < 0.01$ but not $p < 0.05$ or $p < 0.001$. Because threshold selection is ultimately arbitrary, it is important that major conclusions are independent of

parameter choices. We demonstrated that only a small fraction of mouse and rat orthologs respond similarly to TCDD by selecting genes that were statistically significant at $p_{adjusted} < 0.01$ (Figure 3A). To verify this conclusion we selected significance thresholds of $p_{adjusted} < 0.05$ (A) and $p_{adjusted} < 0.001$ (B). In each case the number of common responses ranges from 4-22% of all TCDD-responsive genes.

Supplementary Figure 8: Parameter Sensitivity of P-Value Correlation Analysis

To test if genes with strong evidence (low p-values) for differential expression in one species would have similarly strong evidence (low p-values) in the other we plotted the adjusted p-values for the two species in $\log_{10}$ space. A) When we selected genes that were differentially expressed in either species at a moderate-stringency threshold ($p_{adjusted} < 0.05$) these values are strongly anti-correlated (Spearman's rho = -0.54, $p < 2.2 \times 10^{-16}$). B) When we selected genes that were differentially expressed in either species at a high-stringency threshold ($p_{adjusted} < 0.001$) we again observed a strong anti-correlation (Spearman's rho = -0.43, $p = 5.25 \times 10^{-11}$). Taken together these data suggest that genes that have strong evidence for differential expression in one species are generally likely to have weak or no evidence in the other, and that this conclusion is independent of the p-value threshold used to select genes.

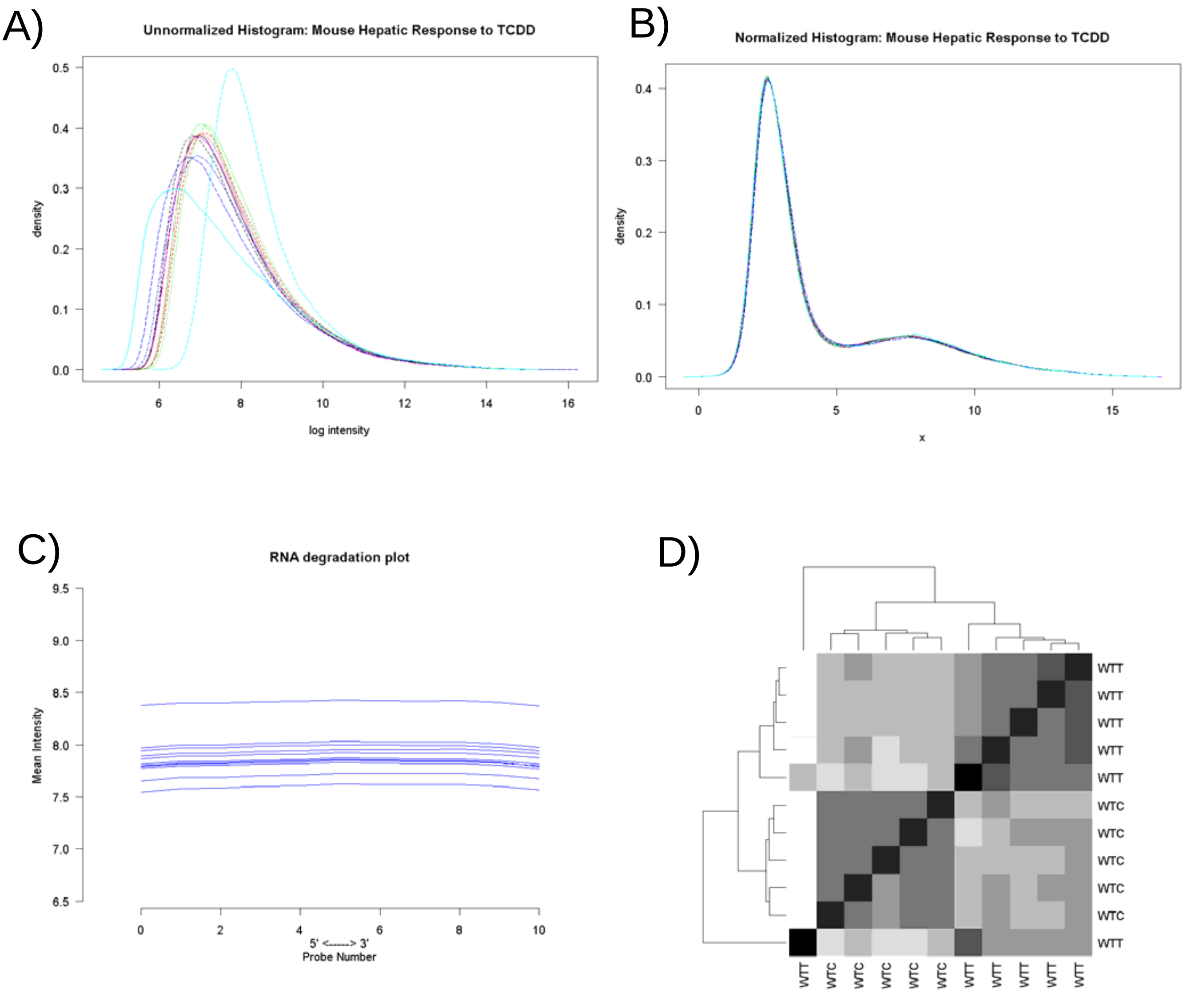

Supplementary Figure 1

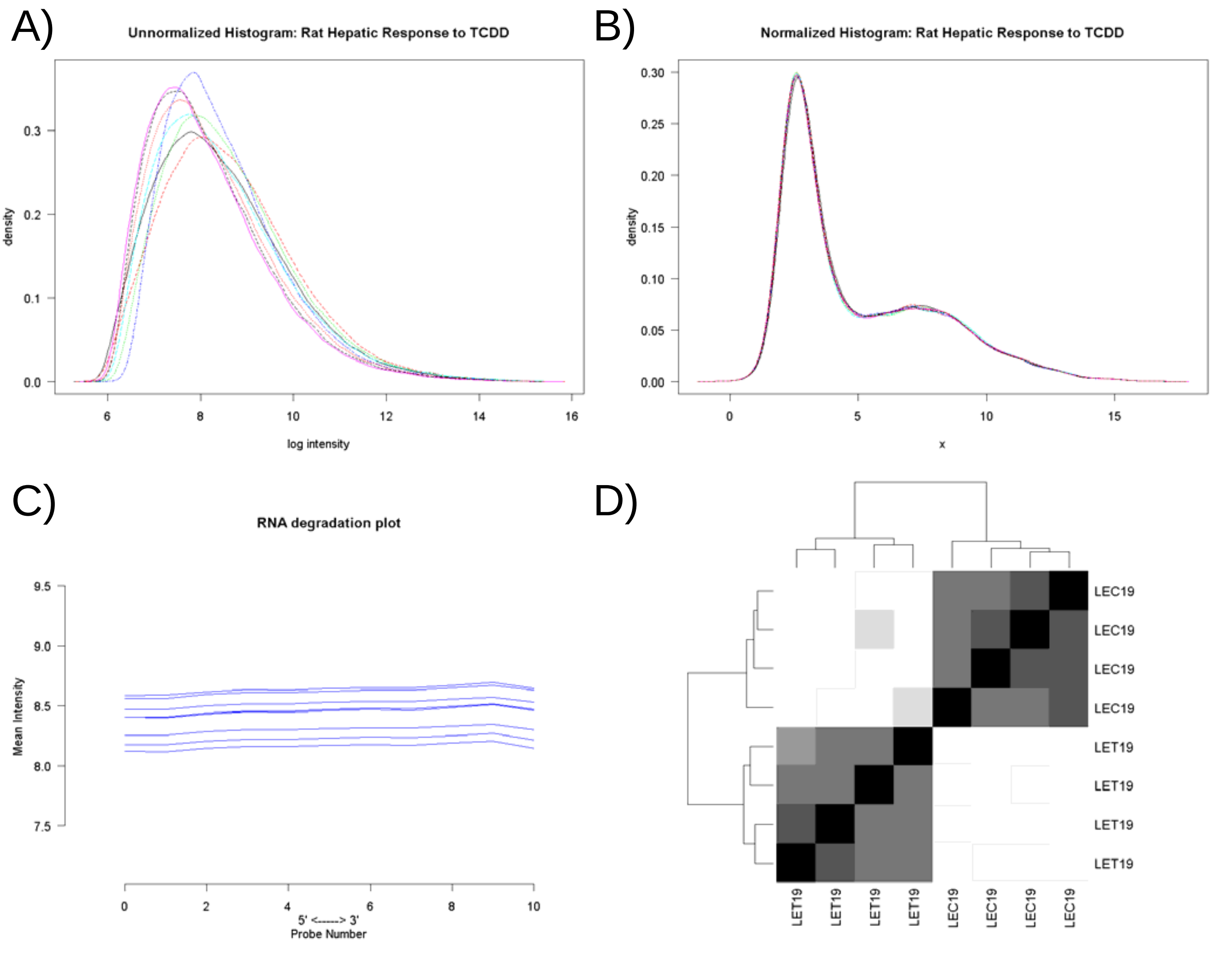

Supplementary Figure 2

A)

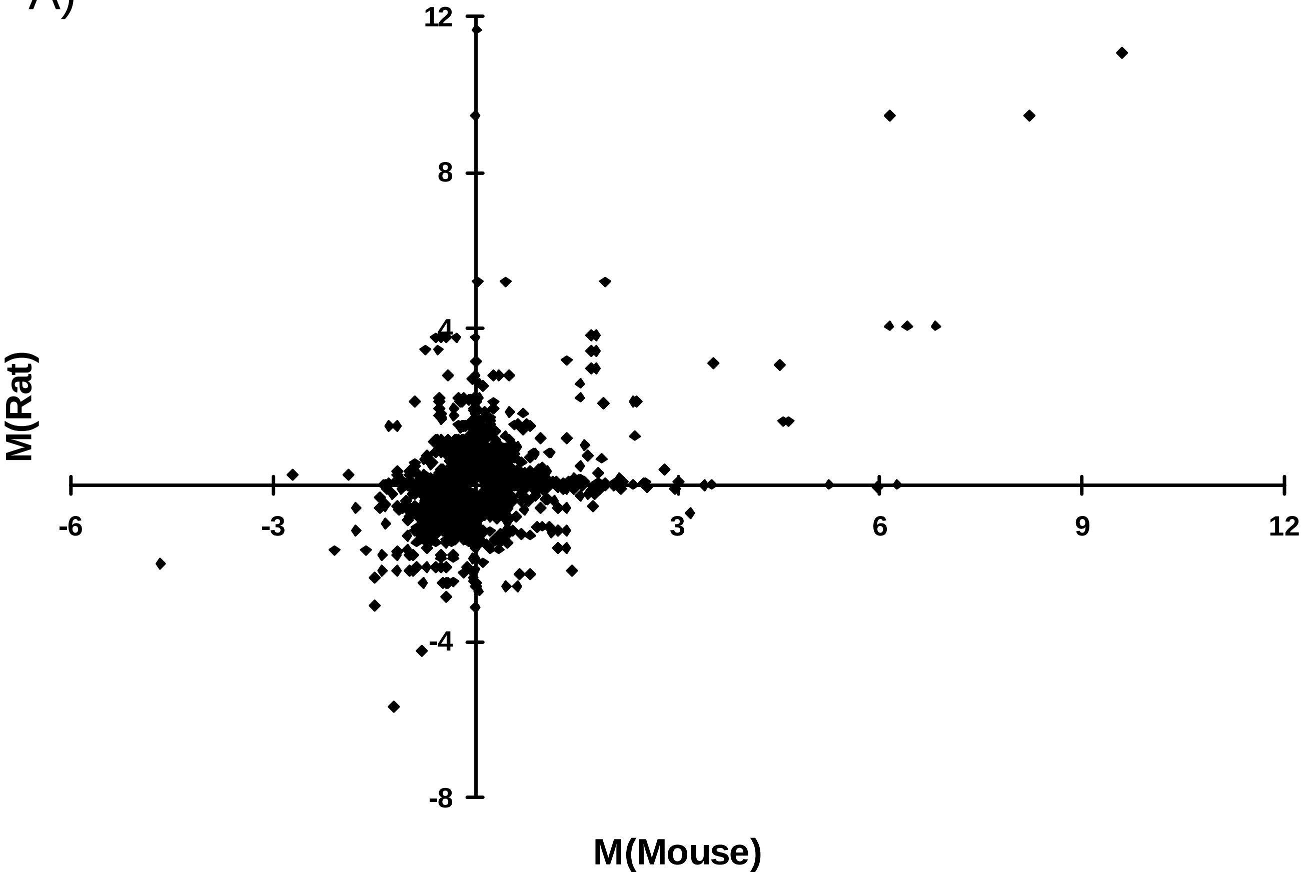

B)

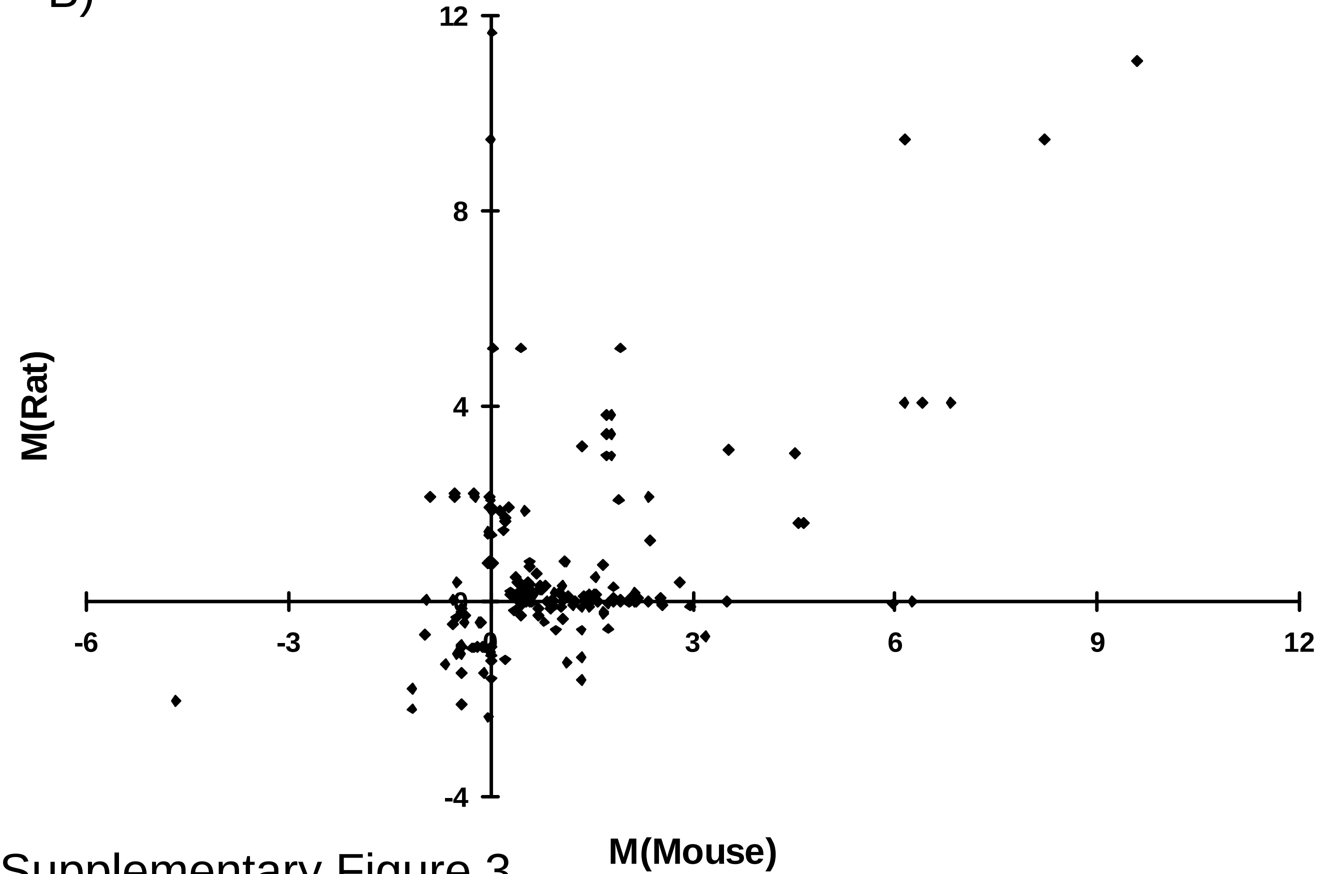

Supplementary Figure 3

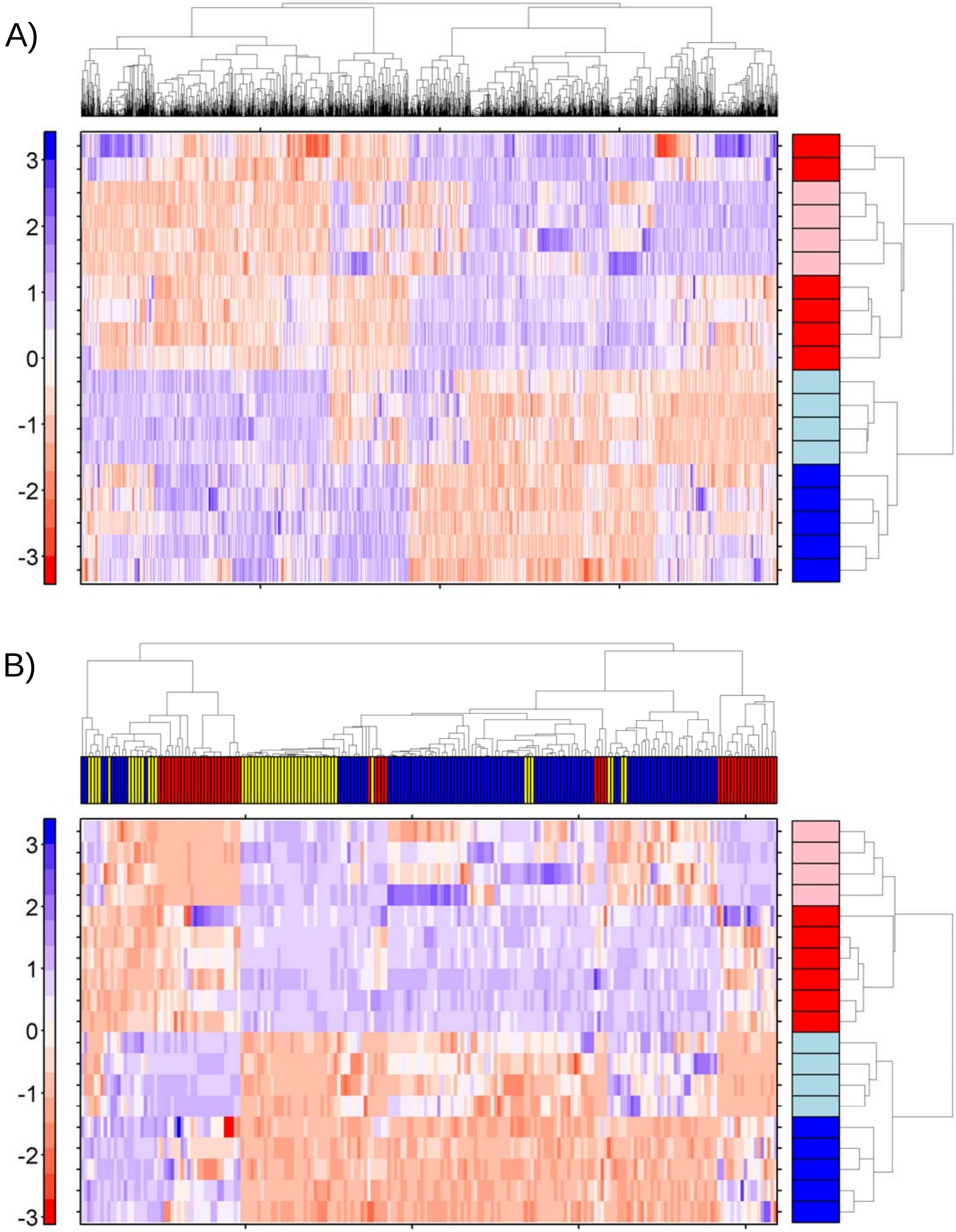

Supplementary Figure 4

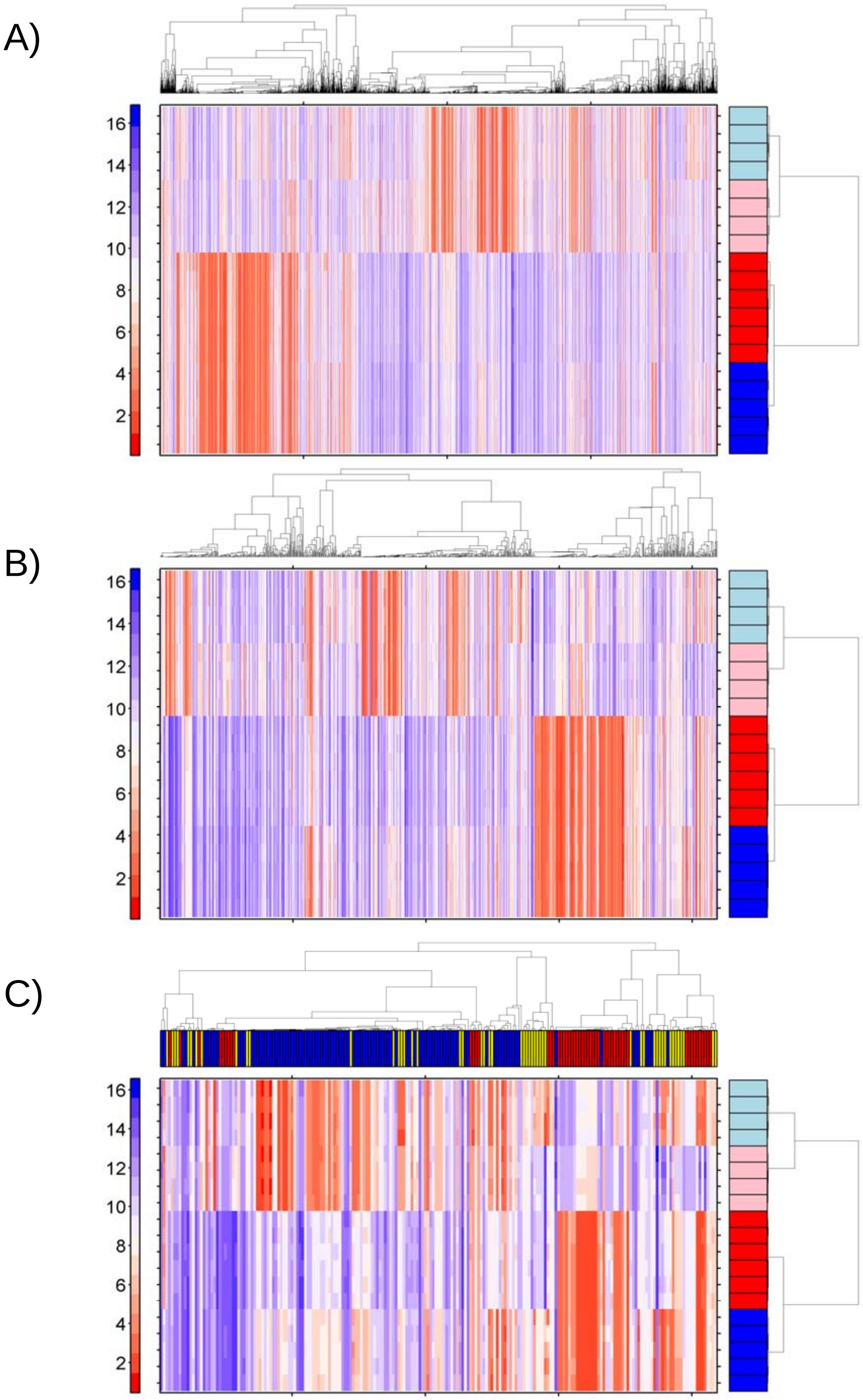

Supplementary Figure 5

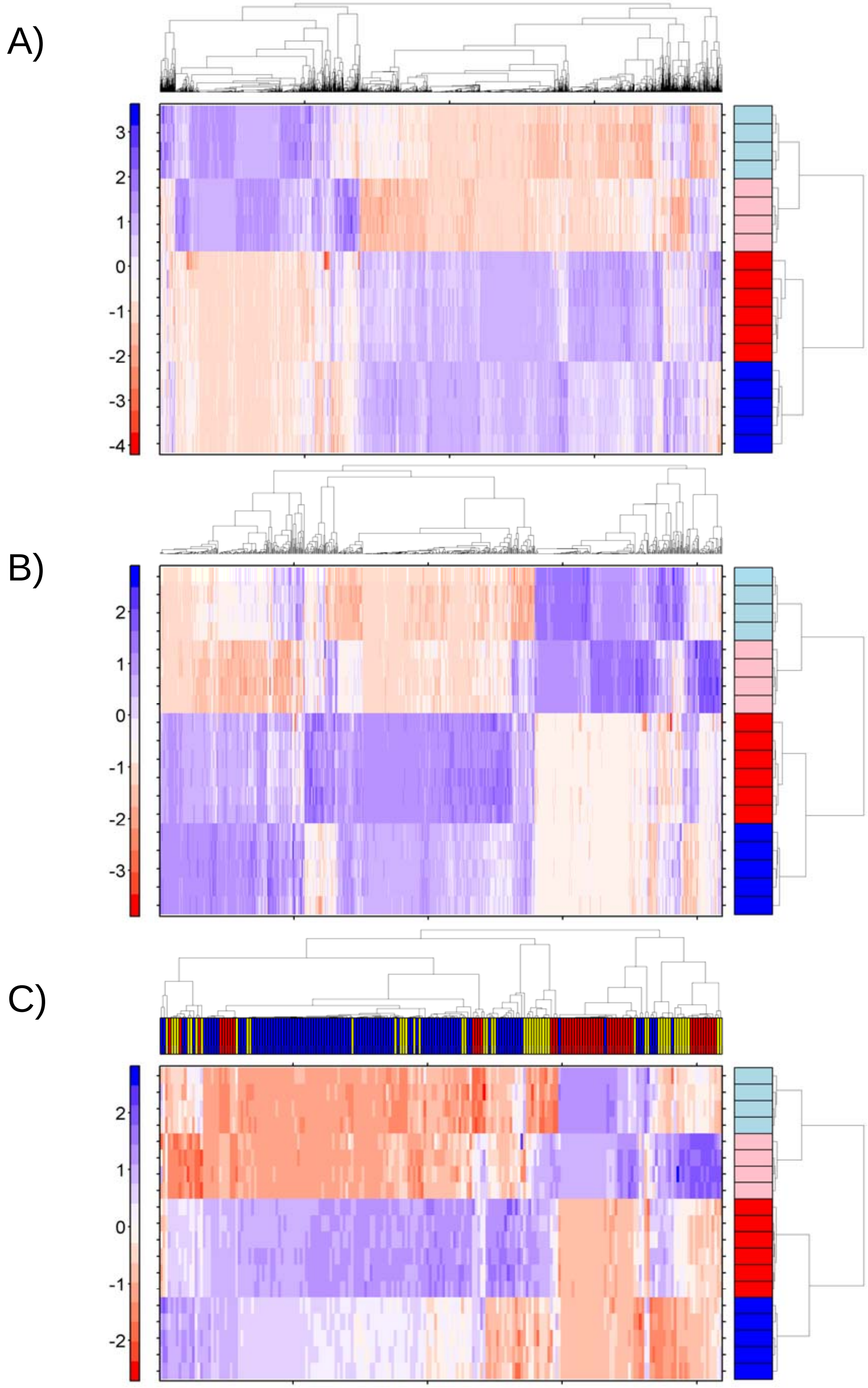

Supplementary Figure 6

A)

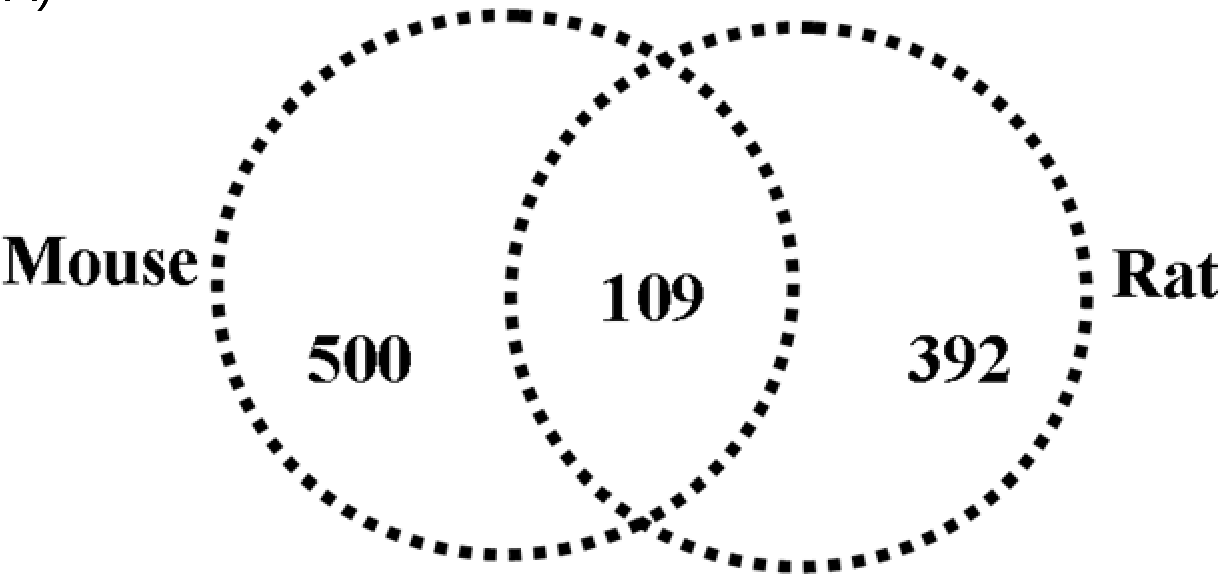

B)

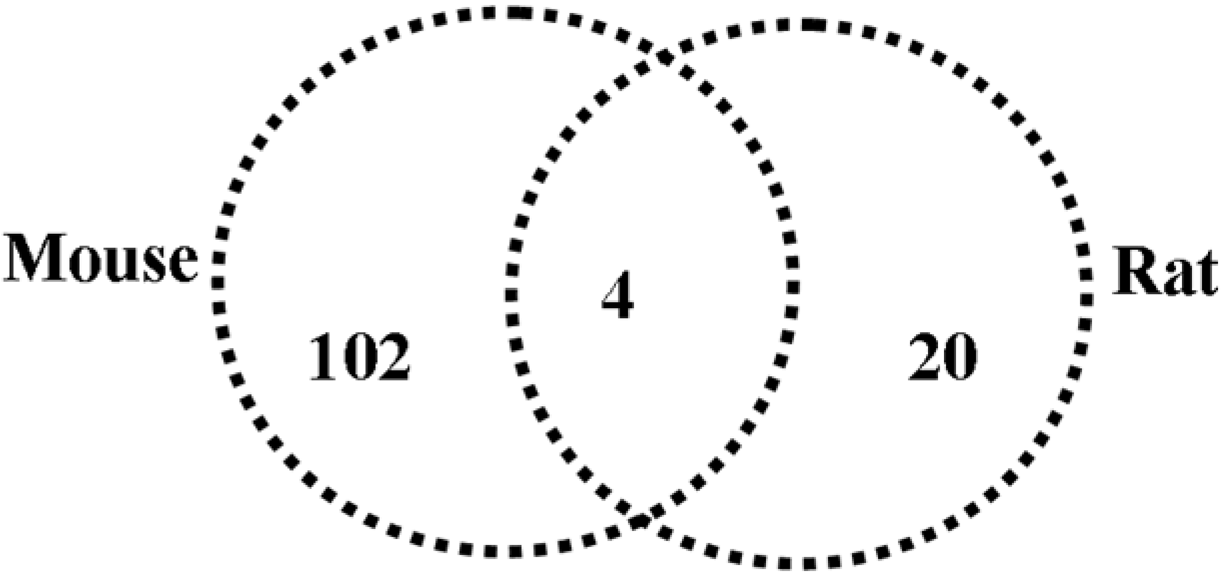

Supplementary Figure 7

A)

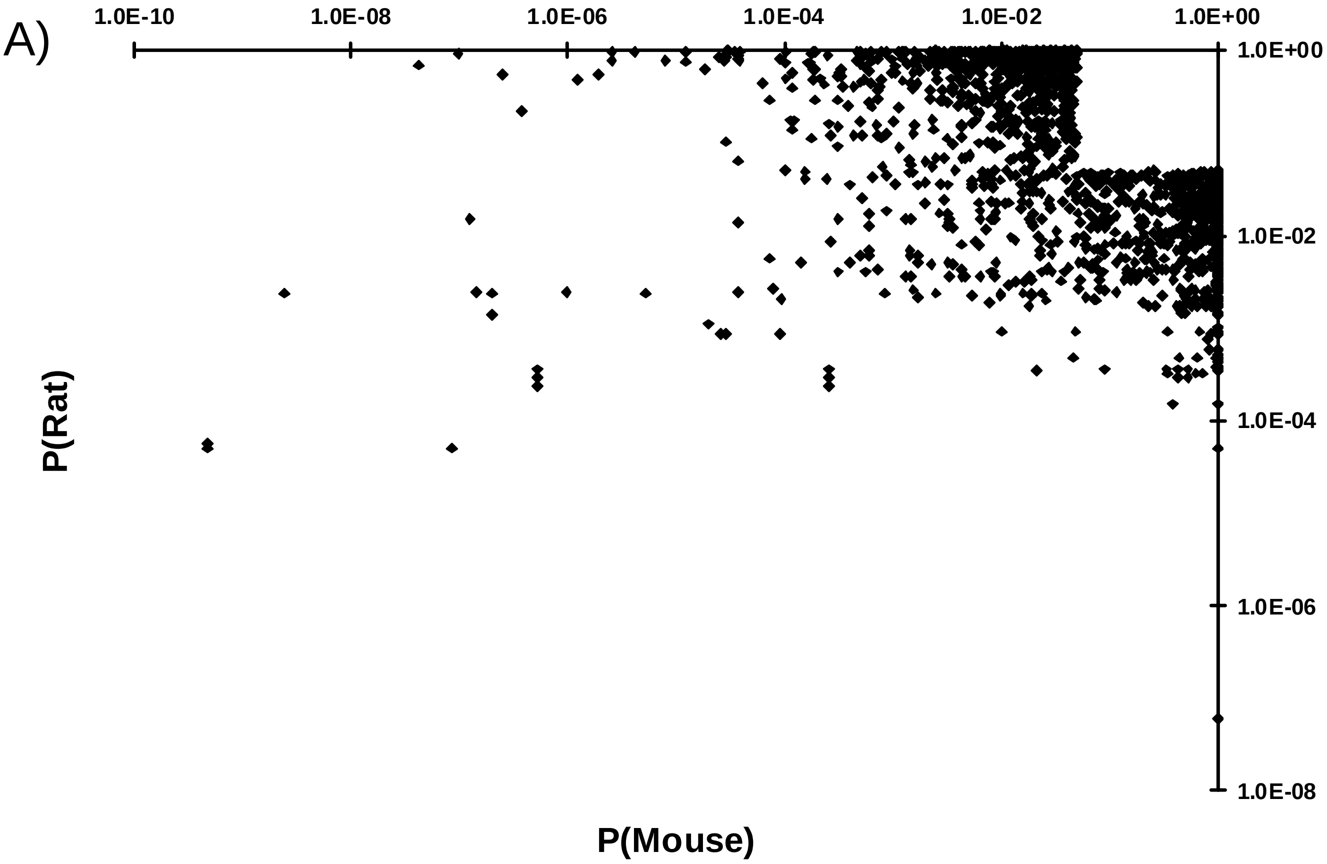

B)

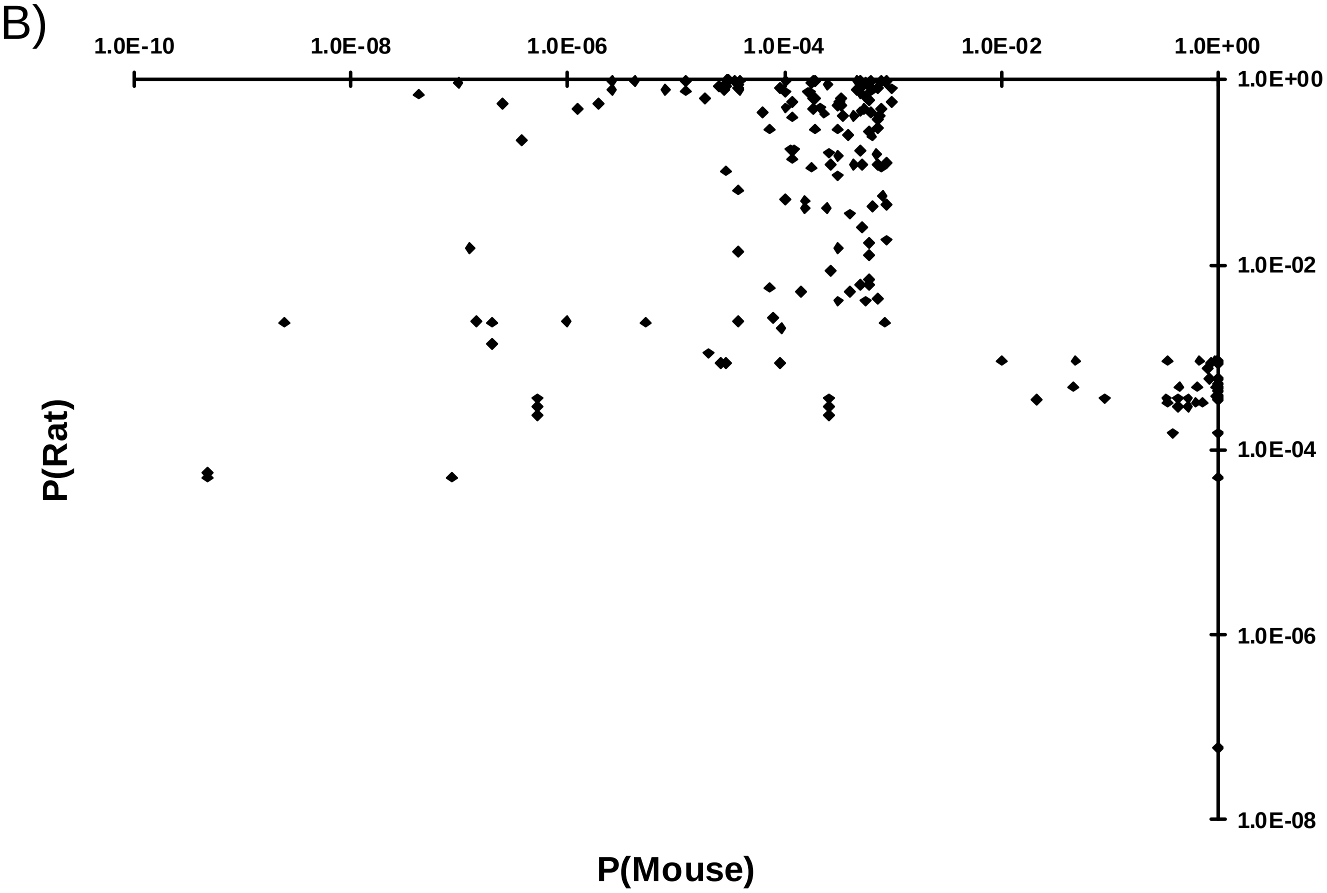

Supplementary Figure 8
